# Methyl 3,3,6,6-tetra­methyl-1,8-dioxo-4,5,7,9-tetra­hydro-2*H*-xanthene-9-carboxyl­ate

**DOI:** 10.1107/S2414314620010184

**Published:** 2020-07-28

**Authors:** Heiner Detert, Laura Kluge, Dieter Schollmeyer

**Affiliations:** a Johannes Gutenberg University Mainz, Department of Chemistry, Duesbergweg 10-14, 55099 Mainz, Germany; Dublin City University, Ireland

**Keywords:** crystal structure, heterocycles, polycyclic system

## Abstract

The title mol­ecule, C_19_H_24_O_5_, is built by annulation of a half-chair cyclo­hexenone and a twist-cyclo­hexenone to a flat 4-*H*-pyrane boat. In the crystal, mol­ecules are connected *via* van der Waals inter­actions and C—H⋯O hydrogen bonds.

## Abstract 2

The title mol­ecule is built by annulation of a half-chair cyclo­hexenone and a twist-cyclo­hexenone to a flat 4-*H*-pyrane boat. In the crystal, mol­ecules are connected *via* van der Waals inter­actions and C—H⋯O hydrogen bonds.

## Graphical abstract

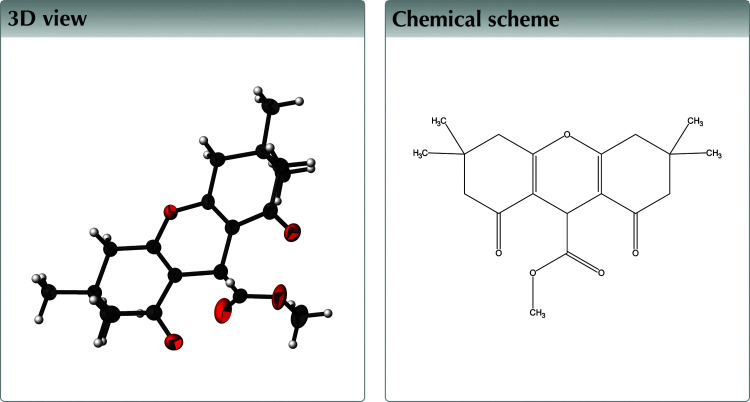

## Structure description

The title compound was obtained as a side product during the formation of methyl meth­oxy(2,6-dioxo-4,4-di­methyl­cyclo­hex­yl)acetate according to the procedure of Grosz & Freiberg (1966 ▸). A similar product (1,2,3,4,5,6,7,8-octa­hydro-3,3,6,6-tetra­methyl-1,8-dioxo-9-xanthenyl acetic acid) was obtained by Gustafsson (1948 ▸) in the condensation of dimedone and glyoxalic acid. The free acid is an isomer of the title compound with a methyl­ene group connecting the heterocyclic unit and carb­oxy­lic acid group. A short route to these compounds is the uncatalysed tandem aldol condensation/elimination/Michael addition/condensation, as discovered by Rohr & Mahrwald (2009 ▸).

**Figure 1:**
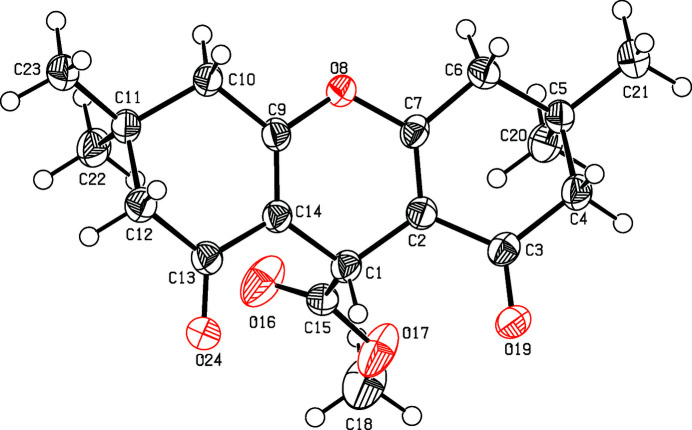
Perspective view of the title compound. Displacement ellipsoids are drawn at the 50% probability level.

The mol­ecule is composed of two di­methyl­cyclo­hexenone units annulated to a central 4*H*-pyrane (Fig. 1 ▸). While the conformation of the latter is a flat boat, one cyclo­hexenone (C2–C7) forms a half-chair and the other (C9–C14) has a twist form. The pyrane boat promotes a folded shape of the mol­ecule, the angle between the mean planes through atoms C1–C3/C6/C7/O8 and O8/C9/C10/C13//C14 being 22.42 (11)°, with maximum deviations from the mean planes at O8 [−0.1046 (18) Å] and C1 [0.051 (3) Å]. The torsion angle of the ester group (O17—C15—C1—C2) is 66.4 (3)°.

**Figure 2:**
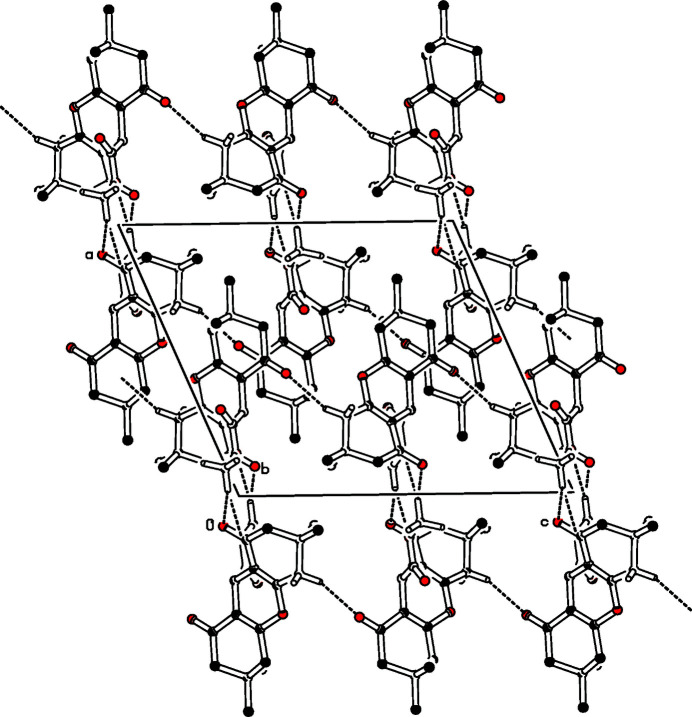
Partial packing diagram of the title compound with a view along the *b* axis. Most of the hydrogen atoms omitted for clarity. Hydrogen bonds are depicted with dashed lines.

Four mol­ecules occupy the monoclinic unit cell, the packing in the cell being dominated by van der Waals inter­actions and hydrogen-bonding inter­actions (Table 1 ▸ and Fig. 2 ▸). The C—H⋯O hydrogen bonds (Steiner, 1996 ▸) C18—H18*A*⋯O17 and C18—H18*A*⋯O19 form a hydrogen-bonded dimer while the C6—H6*B*⋯O24 inter­action connects two mol­ecules related by the *c*-glide plane.

## Synthesis and crystallization

Dimedone (7.01 g, 0.05 mol, 1 eq.) and tri­ethyl­amine (5.05 g, 50 mmol, 6.9 ml, 1 eq.) were dissolved in di­chloro­methane (25 ml) in a 250 ml flask under nitro­gen. Methyl chloro­meth­oxy­acetate (5.9 ml, 6.49 g, 0.525 mol, 1.05 eq.) was added dropwise to the ice-cooled mixture under stirring and the stirring was continued for 75 min at room temperature and a further 3 h under reflux conditions. The solvent was evaporated, methyl *tert*-butyl ether was added to the suspension and triethyl ammonium chloride was removed *via* filtration. The etheral layer was washed with aqueous sodium carbonate and brine, and dried over sodium sulfate. Evaporation of the solvent and chromatography (silica gel, petroleum ether/ethyl acetate = 3/1, *R*_f_ = 3:1) yielded 0.83 g (2.5 mmol, 5%) of the title compound as colourless crystals with m.p. = 474–478 K. The main product yield was 83%. Crystals of the title compound were obtained from a solution in ethyl acetate. IR: 2959, 2875, 1728, 1663, 1368, 1193, 995. ^1^H NMR (300 MHz, CDCl_3_) δ/p.p.m.: 4.47–4.46 (*s*, 1H), 3.68 (*s*, 3H), 2.43 (2*d, 2*2 *gem* H, *J* = 18 Hz), 4H), 2.27 (2*d, 2*2 *gem* H, *J* = 18 Hz), 1.11 (*s*, 12H).

## Refinement

Crystal data, data collection and structure refinement details are summarized in Table 2 ▸.

## Supplementary Material

Crystal structure: contains datablock(s) I, global. DOI: 10.1107/S2414314620010184/gg4005sup1.cif

Structure factors: contains datablock(s) I. DOI: 10.1107/S2414314620010184/gg4005Isup2.hkl

schematic diagram of the reaction. DOI: 10.1107/S2414314620010184/gg4005sup3.tif

Supporting information file. DOI: 10.1107/S2414314620010184/gg4005Isup4.cml

CCDC reference: 2018468

Additional supporting information:  crystallographic information; 3D view; checkCIF report

## Figures and Tables

**Table 1:** Hydrogen-bond geometry (Å, °)

*D*—H⋯*A*	*D*—H	H⋯*A*	*D*⋯*A*	*D*—H⋯*A*
C6—H6*B*⋯O24^i^	0.99	2.50	3.324 (4)	140
C18—H18*A*⋯O17^ii^	0.98	2.53	3.451 (5)	156
C18—H18*A*⋯O19^ii^	0.98	2.41	3.093 (4)	126

Symmetry codes: (i) 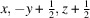 ; (ii) 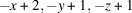 .

**Table 2:** Experimental details

Crystal data	
Chemical formula	C_19_H_24_O_5_
*M*_r_	332.38
Crystal system, space group	Monoclinic, *P*2_1_/*c*
Temperature (K)	120
*a*, *b*, *c* (Å)	13.1494 (10), 9.6899 (6), 14.8185 (13)
β (°)	113.295 (6)
*V* (Å^3^)	1734.2 (2)
*Z*	4
Radiation type	Mo *K*α
μ (mm^−1^)	0.09
Crystal size (mm)	0.22 × 0.11 × 0.06

Data collection	
Diffractometer	Stoe IPDS 2T
No. of measured, independent and observed [*I* > 2σ(*I*)] reflections	8583, 4123, 2784
*R*_int_	0.036
(sin θ/λ)_max_ (Å^−1^)	0.659

Refinement	
*R*[*F* ^2^ > 2σ(*F* ^2^)], *wR*(*F* ^2^), *S*	0.072, 0.185, 1.05
No. of reflections	4123
No. of parameters	222
H-atom treatment	H-atom parameters constrained
Δρ_max_, Δρ_min_ (e Å^−3^)	0.24, −0.30

Computer programs: *X-AREA WinXpose*, *X-AREA Recipe* and *X-AREA Integrate* (Stoe & Cie, 2019 ▸), *SIR2004* (Burla *et al.*, 2005 ▸), *SHELXL2018/3* (Sheldrick, 2015 ▸) and *PLATON* (Spek, 2020 ▸).

## full crystallographic data

### Crystal data

**Table:**

C_19_H_24_O_5_	*F*(000) = 712
*M**_r_* = 332.38	*D*_x_ = 1.273 Mg m^−^^3^
Monoclinic, *P*2_1_/*c*	Mo *K*α radiation, λ = 0.71073 Å
*a* = 13.1494 (10) Å	Cell parameters from 9146 reflections
*b* = 9.6899 (6) Å	θ = 2.6–28.2°
*c* = 14.8185 (13) Å	µ = 0.09 mm^−^^1^
β = 113.295 (6)°	*T* = 120 K
*V* = 1734.2 (2) Å^3^	Plate, colourless
*Z* = 4	0.22 × 0.11 × 0.06 mm

### Data collection

**Table:**

STOE IPDS 2T diffractometer	2784 reflections with *I* > 2σ(*I*)
Radiation source: sealed X-ray tube, 12 x 0.4 mm long-fine focus	*R*_int_ = 0.036
Detector resolution: 6.67 pixels mm^-1^	θ_max_ = 28.0°, θ_min_ = 2.6°
rotation method, ω scans	*h* = −16→17
8583 measured reflections	*k* = −12→12
4123 independent reflections	*l* = −19→19

### Refinement

**Table:**

Refinement on *F*^2^	Primary atom site location: dual
Least-squares matrix: full	Hydrogen site location: inferred from neighbouring sites
*R*[*F*^2^ > 2σ(*F*^2^)] = 0.072	H-atom parameters constrained
*wR*(*F*^2^) = 0.185	*w* = 1/[σ^2^(*F*_o_^2^) + (0.0577*P*)^2^ + 2.4014*P*] where *P* = (*F*_o_^2^ + 2*F*_c_^2^)/3
*S* = 1.05	(Δ/σ)_max_ = 0.002
4123 reflections	Δρ_max_ = 0.24 e Å^−^^3^
222 parameters	Δρ_min_ = −0.30 e Å^−^^3^
0 restraints	

### Special details

**Table:**

Geometry. All esds (except the esd in the dihedral angle between two l.s. planes) are estimated using the full covariance matrix. The cell esds are taken into account individually in the estimation of esds in distances, angles and torsion angles; correlations between esds in cell parameters are only used when they are defined by crystal symmetry. An approximate (isotropic) treatment of cell esds is used for estimating esds involving l.s. planes.
Refinement. Hydrogen atoms attached to carbons were placed at calculated positions and were refined in the riding-model approximation with C–H = 0.95 Å, and with *U*_iso_(H) = 1.2 *U*_eq_(C).

### Fractional atomic coordinates and isotropic or equivalent isotropic displacement parameters (Å^2^)

**Table:**

	*x*	*y*	*z*	*U*_iso_*/*U*_eq_	
C1	0.6910 (2)	0.3522 (3)	0.3759 (2)	0.0290 (5)	
H1	0.715526	0.337644	0.320767	0.035*	
C2	0.7343 (2)	0.2359 (3)	0.4490 (2)	0.0284 (5)	
C3	0.8403 (2)	0.1694 (3)	0.4650 (2)	0.0292 (5)	
C4	0.8801 (2)	0.0564 (3)	0.5415 (2)	0.0327 (6)	
H4A	0.962100	0.056545	0.570806	0.039*	
H4B	0.855649	−0.033835	0.508675	0.039*	
C5	0.8385 (2)	0.0705 (3)	0.6237 (2)	0.0302 (6)	
C6	0.7113 (2)	0.0808 (3)	0.5759 (2)	0.0313 (6)	
H6A	0.679951	−0.010095	0.548221	0.038*	
H6B	0.683107	0.105346	0.626632	0.038*	
C7	0.67398 (19)	0.1865 (3)	0.49633 (19)	0.0270 (5)	
O8	0.56625 (14)	0.22547 (18)	0.47408 (13)	0.0293 (4)	
C9	0.5126 (2)	0.2955 (3)	0.38683 (19)	0.0283 (5)	
C10	0.3910 (2)	0.3002 (3)	0.3590 (2)	0.0301 (6)	
H10A	0.375949	0.313286	0.418829	0.036*	
H10B	0.358002	0.211094	0.328896	0.036*	
C11	0.3362 (2)	0.4177 (3)	0.2862 (2)	0.0312 (6)	
C12	0.3794 (2)	0.4120 (3)	0.2043 (2)	0.0329 (6)	
H12A	0.354823	0.324715	0.167285	0.040*	
H12B	0.347003	0.489267	0.158080	0.040*	
C13	0.5040 (2)	0.4207 (3)	0.2430 (2)	0.0302 (5)	
C14	0.5668 (2)	0.3511 (3)	0.33598 (19)	0.0276 (5)	
C15	0.7354 (2)	0.4908 (3)	0.4252 (2)	0.0307 (6)	
O16	0.68175 (18)	0.5800 (2)	0.4402 (2)	0.0579 (7)	
O17	0.84313 (17)	0.5003 (2)	0.4521 (2)	0.0606 (8)	
C18	0.8958 (3)	0.6248 (3)	0.5039 (3)	0.0611 (11)	
H18A	0.976272	0.616654	0.525273	0.092*	
H18B	0.877556	0.637293	0.561397	0.092*	
H18C	0.869276	0.704400	0.460044	0.092*	
O19	0.89116 (15)	0.19842 (19)	0.41368 (14)	0.0334 (4)	
C20	0.8901 (2)	0.1983 (3)	0.6859 (2)	0.0369 (6)	
H20A	0.971000	0.190656	0.712677	0.055*	
H20B	0.865283	0.204928	0.740014	0.055*	
H20C	0.867042	0.281133	0.644830	0.055*	
C21	0.8723 (2)	−0.0569 (3)	0.6899 (2)	0.0407 (7)	
H21A	0.841283	−0.139699	0.650705	0.061*	
H21B	0.844160	−0.048635	0.741779	0.061*	
H21C	0.953273	−0.063927	0.719617	0.061*	
C22	0.3647 (2)	0.5576 (3)	0.3387 (2)	0.0386 (7)	
H22A	0.332418	0.631853	0.290953	0.058*	
H22B	0.445306	0.568687	0.369190	0.058*	
H22C	0.334574	0.561723	0.389470	0.058*	
C23	0.2108 (2)	0.3990 (3)	0.2434 (2)	0.0386 (7)	
H23A	0.175703	0.475736	0.198843	0.058*	
H23B	0.184611	0.397602	0.296837	0.058*	
H23C	0.191455	0.311806	0.207127	0.058*	
O24	0.55164 (16)	0.4811 (2)	0.19806 (15)	0.0394 (5)	

### Atomic displacement parameters (Å^2^)

**Table:**

	*U*^11^	*U*^22^	*U*^33^	*U*^12^	*U*^13^	*U*^23^
C1	0.0238 (11)	0.0255 (12)	0.0370 (14)	−0.0006 (10)	0.0111 (10)	0.0008 (10)
C2	0.0257 (12)	0.0230 (12)	0.0356 (14)	−0.0012 (10)	0.0110 (11)	−0.0004 (10)
C3	0.0251 (12)	0.0255 (12)	0.0373 (15)	−0.0023 (10)	0.0125 (11)	−0.0049 (11)
C4	0.0301 (13)	0.0271 (13)	0.0409 (16)	0.0045 (11)	0.0142 (12)	0.0013 (11)
C5	0.0253 (12)	0.0290 (13)	0.0340 (14)	0.0027 (10)	0.0095 (11)	0.0048 (11)
C6	0.0294 (12)	0.0268 (12)	0.0375 (15)	−0.0010 (11)	0.0131 (11)	0.0041 (11)
C7	0.0209 (11)	0.0234 (11)	0.0349 (14)	−0.0004 (9)	0.0091 (10)	−0.0030 (10)
O8	0.0231 (8)	0.0295 (9)	0.0348 (10)	0.0014 (7)	0.0109 (7)	0.0041 (8)
C9	0.0268 (12)	0.0237 (12)	0.0326 (14)	0.0025 (10)	0.0100 (10)	0.0026 (10)
C10	0.0240 (12)	0.0266 (13)	0.0385 (15)	0.0018 (10)	0.0110 (11)	0.0034 (11)
C11	0.0251 (12)	0.0267 (13)	0.0402 (15)	0.0024 (10)	0.0113 (11)	0.0050 (11)
C12	0.0279 (12)	0.0315 (13)	0.0380 (15)	0.0011 (11)	0.0115 (11)	0.0021 (11)
C13	0.0297 (12)	0.0256 (12)	0.0351 (14)	0.0012 (10)	0.0125 (11)	0.0017 (11)
C14	0.0244 (11)	0.0229 (12)	0.0344 (14)	0.0005 (10)	0.0103 (10)	0.0008 (10)
C15	0.0262 (12)	0.0258 (12)	0.0396 (15)	−0.0022 (10)	0.0124 (11)	0.0033 (11)
O16	0.0392 (12)	0.0427 (13)	0.094 (2)	−0.0050 (10)	0.0282 (13)	−0.0255 (13)
O17	0.0286 (11)	0.0317 (11)	0.116 (2)	−0.0079 (9)	0.0229 (13)	−0.0240 (13)
C18	0.0410 (17)	0.0309 (16)	0.104 (3)	−0.0120 (14)	0.0204 (19)	−0.0201 (18)
O19	0.0305 (9)	0.0317 (10)	0.0425 (11)	−0.0013 (8)	0.0193 (9)	−0.0026 (8)
C20	0.0311 (13)	0.0402 (16)	0.0355 (15)	0.0013 (12)	0.0089 (12)	−0.0025 (12)
C21	0.0368 (15)	0.0381 (16)	0.0460 (18)	0.0077 (13)	0.0151 (13)	0.0103 (13)
C22	0.0377 (14)	0.0295 (14)	0.0512 (18)	0.0048 (12)	0.0204 (14)	−0.0010 (13)
C23	0.0289 (13)	0.0376 (15)	0.0461 (17)	0.0024 (12)	0.0112 (12)	0.0106 (13)
O24	0.0367 (10)	0.0391 (11)	0.0470 (12)	0.0054 (9)	0.0214 (10)	0.0129 (9)

### Geometric parameters (Å, º)

**Table:**

C1—C14	1.501 (3)	C11—C12	1.532 (4)
C1—C2	1.510 (4)	C11—C22	1.534 (4)
C1—C15	1.530 (4)	C12—C13	1.508 (3)
C1—H1	1.0000	C12—H12A	0.9900
C2—C7	1.337 (3)	C12—H12B	0.9900
C2—C3	1.468 (3)	C13—O24	1.229 (3)
C3—O19	1.228 (3)	C13—C14	1.461 (4)
C3—C4	1.513 (4)	C15—O16	1.191 (3)
C4—C5	1.526 (4)	C15—O17	1.314 (3)
C4—H4A	0.9900	O17—C18	1.450 (4)
C4—H4B	0.9900	C18—H18A	0.9800
C5—C21	1.529 (4)	C18—H18B	0.9800
C5—C20	1.533 (4)	C18—H18C	0.9800
C5—C6	1.541 (3)	C20—H20A	0.9800
C6—C7	1.490 (4)	C20—H20B	0.9800
C6—H6A	0.9900	C20—H20C	0.9800
C6—H6B	0.9900	C21—H21A	0.9800
C7—O8	1.374 (3)	C21—H21B	0.9800
O8—C9	1.381 (3)	C21—H21C	0.9800
C9—C14	1.338 (3)	C22—H22A	0.9800
C9—C10	1.486 (3)	C22—H22B	0.9800
C10—C11	1.538 (4)	C22—H22C	0.9800
C10—H10A	0.9900	C23—H23A	0.9800
C10—H10B	0.9900	C23—H23B	0.9800
C11—C23	1.526 (3)	C23—H23C	0.9800
			
C14—C1—C2	108.7 (2)	C22—C11—C10	110.2 (2)
C14—C1—C15	110.2 (2)	C13—C12—C11	112.6 (2)
C2—C1—C15	110.4 (2)	C13—C12—H12A	109.1
C14—C1—H1	109.2	C11—C12—H12A	109.1
C2—C1—H1	109.2	C13—C12—H12B	109.1
C15—C1—H1	109.2	C11—C12—H12B	109.1
C7—C2—C3	118.7 (2)	H12A—C12—H12B	107.8
C7—C2—C1	120.7 (2)	O24—C13—C14	120.7 (2)
C3—C2—C1	120.5 (2)	O24—C13—C12	122.0 (2)
O19—C3—C2	121.0 (2)	C14—C13—C12	117.2 (2)
O19—C3—C4	121.2 (2)	C9—C14—C13	119.2 (2)
C2—C3—C4	117.7 (2)	C9—C14—C1	121.3 (2)
C3—C4—C5	114.0 (2)	C13—C14—C1	119.4 (2)
C3—C4—H4A	108.8	O16—C15—O17	122.7 (3)
C5—C4—H4A	108.8	O16—C15—C1	125.7 (2)
C3—C4—H4B	108.8	O17—C15—C1	111.6 (2)
C5—C4—H4B	108.8	C15—O17—C18	116.8 (2)
H4A—C4—H4B	107.7	O17—C18—H18A	109.5
C4—C5—C21	109.6 (2)	O17—C18—H18B	109.5
C4—C5—C20	109.8 (2)	H18A—C18—H18B	109.5
C21—C5—C20	108.6 (2)	O17—C18—H18C	109.5
C4—C5—C6	107.8 (2)	H18A—C18—H18C	109.5
C21—C5—C6	109.6 (2)	H18B—C18—H18C	109.5
C20—C5—C6	111.5 (2)	C5—C20—H20A	109.5
C7—C6—C5	111.5 (2)	C5—C20—H20B	109.5
C7—C6—H6A	109.3	H20A—C20—H20B	109.5
C5—C6—H6A	109.3	C5—C20—H20C	109.5
C7—C6—H6B	109.3	H20A—C20—H20C	109.5
C5—C6—H6B	109.3	H20B—C20—H20C	109.5
H6A—C6—H6B	108.0	C5—C21—H21A	109.5
C2—C7—O8	123.0 (2)	C5—C21—H21B	109.5
C2—C7—C6	125.6 (2)	H21A—C21—H21B	109.5
O8—C7—C6	111.4 (2)	C5—C21—H21C	109.5
C7—O8—C9	117.1 (2)	H21A—C21—H21C	109.5
C14—C9—O8	122.5 (2)	H21B—C21—H21C	109.5
C14—C9—C10	125.8 (2)	C11—C22—H22A	109.5
O8—C9—C10	111.7 (2)	C11—C22—H22B	109.5
C9—C10—C11	111.8 (2)	H22A—C22—H22B	109.5
C9—C10—H10A	109.2	C11—C22—H22C	109.5
C11—C10—H10A	109.2	H22A—C22—H22C	109.5
C9—C10—H10B	109.2	H22B—C22—H22C	109.5
C11—C10—H10B	109.2	C11—C23—H23A	109.5
H10A—C10—H10B	107.9	C11—C23—H23B	109.5
C23—C11—C12	110.2 (2)	H23A—C23—H23B	109.5
C23—C11—C22	108.8 (2)	C11—C23—H23C	109.5
C12—C11—C22	109.9 (2)	H23A—C23—H23C	109.5
C23—C11—C10	109.5 (2)	H23B—C23—H23C	109.5
C12—C11—C10	108.1 (2)		
			
C14—C1—C2—C7	−25.2 (3)	O8—C9—C10—C11	159.3 (2)
C15—C1—C2—C7	95.7 (3)	C9—C10—C11—C23	167.9 (2)
C14—C1—C2—C3	151.0 (2)	C9—C10—C11—C12	47.7 (3)
C15—C1—C2—C3	−88.1 (3)	C9—C10—C11—C22	−72.5 (3)
C7—C2—C3—O19	170.0 (2)	C23—C11—C12—C13	−176.3 (2)
C1—C2—C3—O19	−6.3 (4)	C22—C11—C12—C13	63.7 (3)
C7—C2—C3—C4	−5.4 (4)	C10—C11—C12—C13	−56.7 (3)
C1—C2—C3—C4	178.2 (2)	C11—C12—C13—O24	−144.6 (3)
O19—C3—C4—C5	156.8 (2)	C11—C12—C13—C14	36.9 (3)
C2—C3—C4—C5	−27.7 (3)	O8—C9—C14—C13	178.5 (2)
C3—C4—C5—C21	173.5 (2)	C10—C9—C14—C13	−1.7 (4)
C3—C4—C5—C20	−67.3 (3)	O8—C9—C14—C1	−5.9 (4)
C3—C4—C5—C6	54.3 (3)	C10—C9—C14—C1	173.9 (2)
C4—C5—C6—C7	−49.8 (3)	O24—C13—C14—C9	174.9 (3)
C21—C5—C6—C7	−168.9 (2)	C12—C13—C14—C9	−6.5 (4)
C20—C5—C6—C7	70.8 (3)	O24—C13—C14—C1	−0.8 (4)
C3—C2—C7—O8	−168.6 (2)	C12—C13—C14—C1	177.8 (2)
C1—C2—C7—O8	7.7 (4)	C2—C1—C14—C9	24.4 (3)
C3—C2—C7—C6	9.2 (4)	C15—C1—C14—C9	−96.7 (3)
C1—C2—C7—C6	−174.5 (2)	C2—C1—C14—C13	−160.0 (2)
C5—C6—C7—C2	20.1 (4)	C15—C1—C14—C13	78.9 (3)
C5—C6—C7—O8	−161.9 (2)	C14—C1—C15—O16	7.8 (4)
C2—C7—O8—C9	13.7 (3)	C2—C1—C15—O16	−112.2 (3)
C6—C7—O8—C9	−164.4 (2)	C14—C1—C15—O17	−173.5 (2)
C7—O8—C9—C14	−14.7 (4)	C2—C1—C15—O17	66.4 (3)
C7—O8—C9—C10	165.5 (2)	O16—C15—O17—C18	1.4 (5)
C14—C9—C10—C11	−20.5 (4)	C1—C15—O17—C18	−177.2 (3)

### Hydrogen-bond geometry (Å, º)

**Table:**

*D*—H···*A*	*D*—H	H···*A*	*D*···*A*	*D*—H···*A*
C6—H6*B*···O24^i^	0.99	2.50	3.324 (4)	140
C18—H18*A*···O17^ii^	0.98	2.53	3.451 (5)	156
C18—H18*A*···O19^ii^	0.98	2.41	3.093 (4)	126

Symmetry codes: (i) *x*, −*y*+1/2, *z*+1/2; (ii) −*x*+2, −*y*+1, −*z*+1.
