# Supplementary figures and images for: Methyl 3,3,6,6-tetra­methyl-1,8-dioxo-4,5,7,9-tetra­hydro-2H-xanthene-9-carboxyl­ate
Source: IUCrdata. 2020 Jul 28;5(Pt 7):x201018. doi: 10.1107/S2414314620010184 (PMC9462258; doi:10.1107/S2414314620010184)

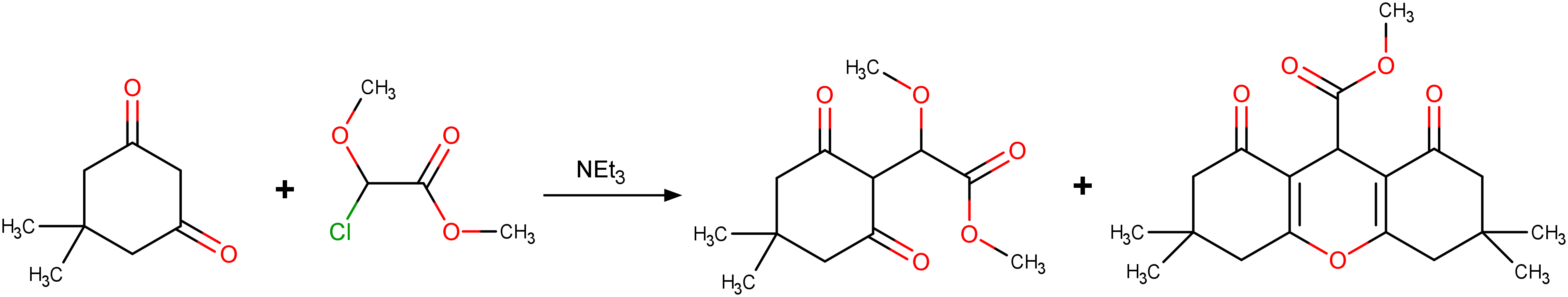

Supplement: Supplementary file 3 [file x-05-x201018-sup3.tif]
